# Prevalence and treatment of mitochondrial diabetes in Southwest Finland

**DOI:** 10.1007/s40200-026-01964-x

**Published:** 2026-06-05

**Authors:** Nelli Rindell, Heidi Immonen, Mika H. Martikainen

**Affiliations:** 1 Clinical Neurosciences, University of Turku, Turku, Finland; 2 Department of Medicine, Turku University Hospital, Turku, Finland; 3 Turku University Hospital, Neurocenter, Turku, Finland; 4 Research Unit of Clinical Neuroscience, Neurology, University of Oulu, Oulu, Finland; 5 Neurocenter and Medical Research Centre, Oulu University Hospital, Oulu, Finland; 6 Department of Neurology, Oulu University Hospital, P.O. Box 10, OYS, Oulu, FIN-90029 Finland

**Keywords:** Diabetes, Mitochondrial disease, Mitochondrial diabetes, Type 1 diabetes, Type 2 diabetes, Gene variant

## Abstract

**Purpose:**

Diabetes mellitus (DM) is a common manifestation of mitochondrial disease, typically associated with the mitochondrial DNA (mtDNA) variant m.3243A>G. We investigated the clinical features, treatment, and epidemiology of mitochondrial DM in the region of Southwest Finland.

**Methods:**

Electronic medical records at Turku University Hospital were searched for patients assigned ICD-10 codes E13.0–E13.9 during 2000–2022. Among 1004 screened individuals, nine patients with genetically confirmed mitochondrial diabetes were identified. Eight additional genetically confirmed patients were included from an ongoing mitochondrial disease research project, resulting in a cohort of 17 patients. The clinical characteristics and DM treatment of the patients were obtained from medical records.

**Results:**

We identified 17 patients with mitochondrial DM. Mean age at diagnosis of DM was 35 years (range 11 to 60 years). Most patients with mitochondrial DM had hearing impairment (14/17). Insulin treatment was typically initiated 3.5 years after the diagnosis of DM. Only six (35%) patients had HbA1c below 7.0% (53 mmol/mol). The prevalence of mitochondrial DM in the region of Southwest Finland in the end of 2022 was 2.7/100,000 and annual incidence during the study period 0.14/100,000.

**Conclusions:**

The onset of non-autoimmune diabetes in young adult age, particularly when associated with hearing impairment, suggests possible mitochondrial DM. Recognition of mitochondrial diabetes is essential for optimal management and complication prevention.

## Introduction

Mitochondria are organelles found in all eukaryotic cells, whose main function is the aerobic production of adenosine triphosphate (ATP) for the energy needs of cells by oxidative phosphorylation [1]. Mitochondrial diseases are a group of genetic diseases in which the mitochondrial energy production is impaired [2]. The most common deleterious mtDNA variant is m.3243A>G in the mtDNA leucine tRNA gene [3]. The associated mitochondrial disease typically manifests in metabolically highly active organs, such as the endocrine pancreas and inner ear, as well as the retina, muscles, kidneys and brain [4, 5]. Well known phenotypes associated with the m.3243A>G variant include maternally inherited diabetes and deafness (MIDD) and the mitochondrial encephalomyopathy, lactic acidosis and stroke-like episodes (MELAS) syndrome [6].

The situation in which diabetes mellitus (DM) is a manifestation of mitochondrial disease is called mitochondrial DM. The most common cause of mitochondrial DM is m.3243A>G [7, 8]. In mitochondrial DM, the glucose metabolism disorder is caused by both impaired insulin secretion by pancreatic beta cells and insulin resistance in muscles [4, 9]. Approximately 1% of DM is estimated to be related to mitochondrial disease [4, 10]. Also, in the Southwest Finland region, approximately 1% of young adult-onset DM is related to the m.3243A>G variant [11]. The prevalence of mitochondrial disease associated with the m.3243A>G variant in the adult population of Southwest Finland is approximately 4.2/100,000 [12]. Mitochondrial DM is typically diagnosed in young adulthood, on average at age of 37 years (range 11–68 years) [4, 10]. Although there are previous publications on the prevalence and clinical features of mitochondrial DM, the treatment of mitochondrial DM has been studied relatively little. Because of features such as high prevalence of diabetic kidney disease [13] and recommendation to avoid metformin [14], identification of DM as caused by mitochondrial disease has important implications for clinical care. Correct diagnosis also enables the detection of other manifestations potentially related to mitochondrial disease such as hearing loss, heart disease, and stroke-like episodes [5]. This study aimed to assess the prevalence, clinical characteristics, and treatment of mitochondrial diabetes in the region of Southwest Finland.

## Methods

### Study design and setting

This retrospective observational register-based study was conducted at Turku University Hospital (TUH, Turku, Finland) in the region of Southwest Finland. Since the diagnostics and treatment of genetic DM in all Southwest Finland are concentrated in TUH, the data on diagnosed and treated patients in TUH describe well the situation for the entire region.

### Patient identification

In early 2023, we searched the TUH electronic patient records to identify those individuals who had received at least one of the ICD-10 diagnosis codes E13.0-E13.9 during the years 2000–2022. ICD-10 codes E13.0–E13.9 correspond to ‘other specified diabetes mellitus’ and its subcategories. We then scrutinised the individual patient data entries in TUH electronic medical record system of these individuals to identify those who had been genetically confirmed to have any form of monogenic DM or mitochondrial DM. In addition to the patients identified in this database search, mitochondrial DM patients previously identified in an on-going clinical and epidemiological research project on mitochondrial disease at TUH were also included.

### Genetic confirmation and definition of mitochondrial diabetes

The confirmed diagnosis of mitochondrial disease was here defined as a clinical phenotype compatible with mitochondrial disease together with a confirmed pathogenic mtDNA variant detected in the patient sample (either in white blood cell or muscle derived mtDNA). Mitochondrial DM was defined as a DM diagnosed in a patient with clinically manifest mitochondrial disease and with genetic evidence of a confirmed pathogenic mtDNA variant. Data on mtDNA heteroplasmy levels were mostly not available. In all patients, the diagnosis was confirmed by genetic testing, either by Sanger sequencing of the mtDNA or by next generation sequencing (NGS) panels that included mtDNA. In most cases, white blood cell mtDNA was used for analysis. Genetic testing was performed either as in-house genetic service at TUH, or externally via several genetic diagnostic services.

### Data collection

From all identified patients with mitochondrial DM, relevant clinical details were collected from the TUH electronic medical records. These included age, gender, weight, height, time of DM diagnosis, time when DM was diagnosed as mitochondrial, DM medication, blood pressure medication, cholesterol medication, age at which insulin was started, daily insulin doses, use of a glucose sensor, use of an insulin pump, recent laboratory values (latest values within one year from data collection) incl. HbA1c, C-peptide, GAD-antibody, urine albumin to creatine ratio, glomerular filtration rate, most recent information on blood pressure level, possible hearing loss and age when hearing aids were used, micro- and macrovascular complications of DM, as well as the main clinical features of the mitochondrial disease based on clinical notes. BMI values are closest available measurements relative to diagnostic stage. Retinopathy classification was based on ophthalmological assessment or diabetes physician assessment of retinal fundus photographs. Neuropathy was recorded if a clinical diagnosis of neuropathy or diabetic neuropathy was documented in the medical records. Nephropathy was defined as a urine albumin-to-creatinine ratio greater than 3 mg/mmol in a spot urine sample or an estimated glomerular filtration rate (eGFR) less than 60 ml/min/1.73 m². In most patients, renal involvement was based on repeated laboratory findings, reduced eGFR, or an established clinical diagnosis documented in the records. However, in some patients, the clinical diagnosis of nephropathy had been made in routine care on the basis of a single elevated urine albumin-to-creatinine ratio measurement. The C-peptide values reported in this study were stimulated measurements, performed together with a simultaneous plasma glucose measurement. For interpretation, the simultaneous glucose concentration was required to be at least 7 mmol/l. The local reference range for stimulated C-peptide was 0.70–1.60 nmol/l. The most recent C-peptide values were measured in some cases at the time of diagnosis and in some cases at a later stage. In the available medical records, the diabetes-associated autoantibody testing referred to GAD antibodies. Insulin autoantibodies were not systematically measured and were therefore not included in the analysis. According to the local laboratory reference range, GAD antibody values < 5 U/ml were considered negative, values 5–9 U/ml borderline, and values ≥ 10 U/ml positive.

### Incidence and prevalence estimates

The estimates for annual incidence and prevalence of mitochondrial DM in Southwest Finland were calculated based on the population data for Southwest Finland available from Statistics Finland’s website (https://pxdata.stat.fi/PxWeb/pxweb/en/StatFin/StatFin__vaerak/statfin_vaerak_pxt_11ra.px/table/tableViewLayout1/). Southwest Finland is a hospital district in Southwestern Finland with a population of 485,567 at the end of 2022. The region includes both urban and rural municipalities, with Turku as the largest city. TUH is the tertiary referral hospital for the region, and diagnostics and specialist care for genetic diabetes are centralized there. The 95% confidence intervals for the prevalence rates were calculated using the Poisson distribution.

### Research permission

This study was part of a larger research project on mitochondrial disease for which a research permit of TUH was obtained (T04/016/16).

## Results

### Cohort identification

**Fig. 1:**
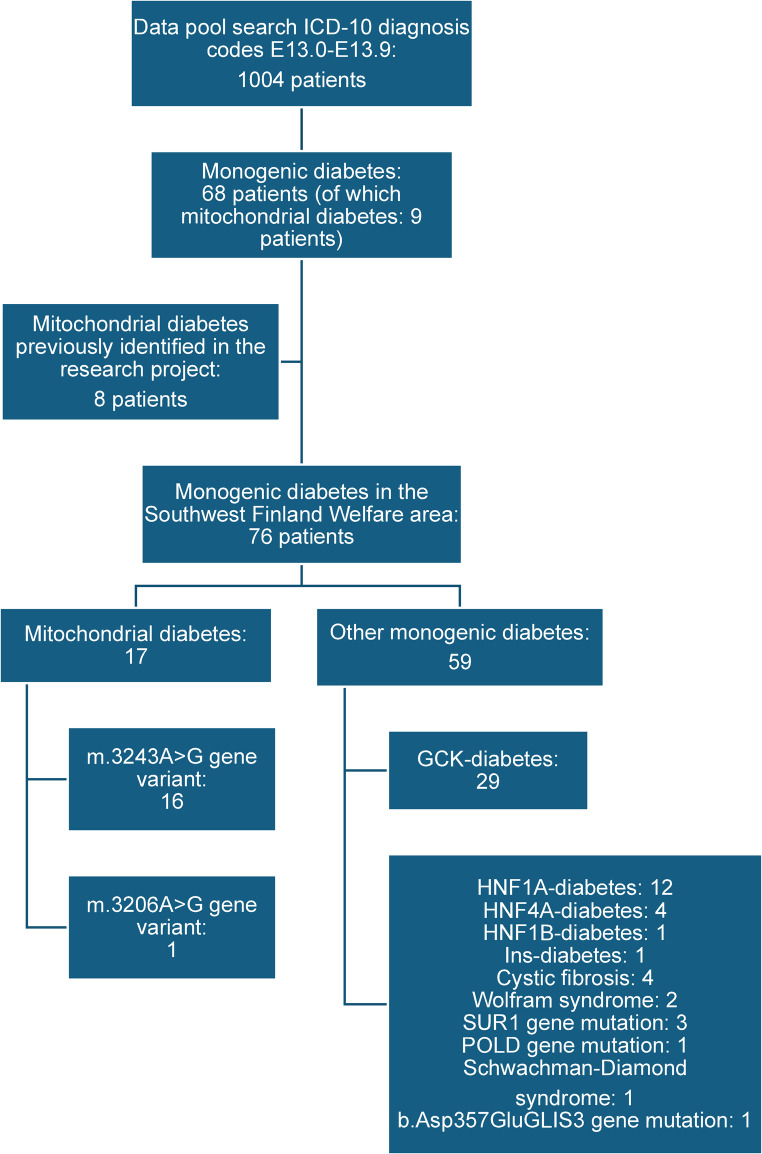
Flowchart of the identification of patients with mitochondrial DM

The ICD-10 diagnosis search identified 1004 patients with at least one of the ICD-10 diagnosis codes E13.0-E13.9. With detailed analysis of patient data, we found 68 patients with mitochondrial DM or some monogenic DM; 9 (13%) of these had mitochondrial DM. In addition, we included eight previously identified patients with mitochondrial DM for whom other ICD-10 codes for DM were used (Fig. 1). All these individuals were also diagnosed during the period of 2000–2022. Finnish population is still comparatively homogenous, despite increasing immigration in the past three decades. In the end of year 2022, 10% of the inhabitants of the region of Southwest Finland were of foreign background, the same proportion as in the whole country (Statistics Finland: https://pxdata.stat.fi/PxWeb/pxweb/en/StatFin/StatFin__vaerak/statfin_vaerak_pxt_11rt.px/). All patients reported in this study were of White European (Finnish) ethnicity.

### Cohort characteristics

In total, *N* = 17 (8 women, 9 men) patients with mitochondrial DM were thus included (Table 1). Three pairs of patients were related: one mother and son, and two pairs of siblings. Four of the patients had died before the study. Among the four deceased individuals, three had died from complications of stroke-like episodes; in one, the cause of death could not be determined based on hospital records. Sixteen (94%) had the m.3243A>G variant. One patient had DM caused by the rarer m.3260A>G gene variant. DM was diagnosed at an average age of 35 years (range 11 to 60 years). The median delay from the diagnosis of DM to the identification of its mitochondrial aetiology was five years (range 2 months–20 years). One patient had been diagnosed with mitochondrial disease before the onset of DM. T1D-associated autoantibodies were tested in 11 out of 17 patients (65%). Of these, 9 tested negative, while 2 showed marginally elevated levels (patient 3: 11 IU/mL; patient 15: 52 IU/mL). None of the patients whose weight was known was overweight (mean body mass index was 21.9 kg/m2). Fourteen patients (14/17, 82%) also had hearing impairment. Among them, twelve had been diagnosed with the hearing impairment prior to their diabetes diagnosis. In one case, diabetes was diagnosed before the onset of hearing loss, although the specific mitochondrial etiology was not yet identified. Conversely, one patient received a diagnosis of mitochondrial disease before developing hearing impairment, as their clinical diabetes manifested later. Ten patients (59%) used a hearing aid, and the hearing aid was introduced at an average age of 38 years.

**Table 1:** Diagnostics and clinical features of patients with mitochondrial DM

Patient	Age (y)	Sex	DM-dg age (y)	Mito-DM dg age (y)	Cause of genetic testing	Insulin treatment	HbA1c (% (mmol/ mol))	C-peptide (nmol/l)	Hearing loss	Hearing aid, age (y)	BMI
P1	64	F	45	60	Symptoms	Yes	7.5 (58)	N/A	Yes	44	22.9
P2	65	F	48	53	Symptoms	Yes	7.1 (54)	N/A	No	–	26.9
P3	33	F	24	24	Symptoms and family history	Yes	7.1 (54)	2.4	Yes	–	23.4
P4	26	M	22	22	Symptoms	Yes	7.4 (57)	0.46	Yes	23	20.8
P5	59 †	M	30	50	Symptoms	Yes	7.7 (61)	N/A	Yes	43	18.4
P6	57	F	52	54	Symptoms	Yes	6.9 (52)	3.5	Yes	50	21.4
P7	54	M	34	50	Symptoms and family history	No	7.2 (55)	2.1	Yes	–	23.7
P8^a^	77	F	60	72	Symptoms and family history	No	6.5 (47)	N/A	Yes	57	15.4
P9^a^	46	M	35	41	Symptoms	Yes	8.1 (65)	1.4	Yes	29	24.0
P10	30	M	26	26	Symptoms	No	5.5 (37)	N/A	Yes	11	22.3
P11^b^	27	M	25	26	Symptoms	Yes	6.0 (42)	0.66	Yes	–	23.6
P12^b^	30	M	27	27	Family history	Yes	6.0 (42)	0.81	No	–	24.7
P13^c^	27 †	F	20	10	Symptoms	Yes	6.7 (50)	0.78	Yes	–	19.6
P14	68	F	44	51	Symptoms	Yes	10 (86)	0.30	Yes	40	20.8
P15^c^	24	M	11	11	Family history	Yes	8.7 (72)	N/A	No	–	18.4
P16	66 †	F	49	61	Symptoms	Yes	8.4 (68)	0.69	Yes	39	21.7
P17	63 †	M	40	55	Symptoms	Yes	8.0 (64)	0.31	Yes	49	25.0

*DM-dg* diagnosis of diabetes, *M *male,* Mito-DM* *dg* diagnosis of mitochondrial diabetes, *F* female, *N/A* data not available, *Y* years, *BMI* values are closest available BMI relative to diagnostic stage, *HbA1c* values are those determined within one year of the study. Related individuals are indicated with matching superscript letters. ^a^mother and son; ^b^siblings; ^c^siblings.

### Treatment of diabetes

Insulin was used for the treatment of DM in 14 patients (82%). Glucose sensor use was reported in 11 (65%), while none used an insulin pump. Among the oral medications, four patients used a gliptin, three patients used a SGLT2 inhibitor in addition to insulin, and one patient used metformin. The mean of the most recent HbA1c values (measured within the last year from the time of the study) was 7.4% (57 mmol/mol) (standard deviation 12 mmol/mol). The details of insulin treatment and other DM medications used are presented in Table 2.

**Table 2:** Treatment of mitochondrial DM in the studied patients

Patient	Current medication for DM	Insulin dosage (IU/kg)	Delay from DM-dg to insulin treatment (y)	All DM medications*	Glucose sensor in use
P1	Insulin, dapagliflozin	0.69	14	Metformin, vildagliptin, dapagliflozin, insulin	Yes
P2	Insulin, dapagliflozin, linagliptin	0.31	8	Sitagliptin, linagliptin, dapagliflozin, insulin	No
P3	Insulin	0.46	6	Linagliptin, insulin	Yes
P4	Insulin	0.47	0	Insulin	Yes
P5	Insulin	0.98	0	Insulin	Yes
P6	Insulin, dapagliflozin	0.23	3	Metformin, dapagliflozin, insulin	Yes
P7	–	–	–	Metformin, glimepiride, sitagliptin, linagliptin, empagliflozin, dapagliflozin	Yes
P8	–	–	–	Metformin	No
P9	Insulin, linagliptin	0.67	0	Metformin, sitagliptin, linagliptin, dapagliflozin, empagliflozin, insulin	Yes
P10	–	–	–	–	No
P11	Insulin	0.53	0	Insulin	Yes
P12	Insulin, sitagliptin	0.56	0	Insulin, sitagliptin	No
P13	Insulin	1.3	0	Insulin	Yes
P14	Insulin	0.38	4	Metformin, rosiglitazone, glimepiride, insulin	Yes
P15	Insulin	0.84	0	Insulin	Yes
P16	Insulin, metformin, sitagliptin	0.40	9	Metformin, glimepiride, glitazone, sitagliptin, insulin	No
P17	Insulin	0.35	5	Insulin	No

*DM* diabetes mellitus

### Cardiovascular risk factors and complications

Nine (53%) patients used a statin at some point, and seven of them (78%) had a history of some statin-attributed muscle symptoms. In these patients, statin treatment had either been modified or discontinued due to adverse effects. Microvascular complications of DM included nephropathy in six patients (35%). The blood pressure and cholesterol medications as well as the diagnosed DM complications are presented in Table 3.

**Table 3:** Treatment of hypertension and hypercholesterolemia and complications of DM in people with mitochondrial DM

Patient	BP medication	BP (mmHg)	Cholesterol medication	Latest U-albcre (mg/mmol)		Latest GFR (ml/min/1.73m^2^)		Retinopathy	Neuropathy	
P1	ARB, CCB	124/75	Ezetimibe		< 0.4		98	Yes		N/A
P2	ACE inhibitor, CCB, beta-blocker	139/82	Ezetimibe		37.1		36	No		Yes
P3	No	128/83	Atorvastatin		< 0.4		115	No		N/A
P4	No	123/77	No		< 0.4		128	No		N/A
P5	No	120/74	No		1.1		147	No		N/A
P6	No	146/95	Ezetimibe, rosuvastatin		< 0.4		61	No		N/A
P7	No	124/87	No		< 0.4		99	No		N/A
P8	No	118/73	Ezetimibe		3.4		74	No		N/A
P9	ACE inhibitor, CCB, beta-blocker	127/92	Atorvastatin		1.1		68	No		N/A
P10	No	133/80	No		N/A		100	No		N/A
P11	No	148/99	No		0.6		115	No		N/A
P12	No	124/81	No		0.7		141	No		N/A
P13	ACE inhibitor, beta-blocker	128/90	No		9.9		59	No		N/A
P14	ARB, CCB	129/68	Ezetimibe, atorvastatin		9.6		64	Yes		N/A
P15	ACE inhibitor	130/80	No		9.2 (4 years ago)		127	Yes		N/A
P16	ARB, hydrochlorothiazide	122/70	Simvastatin		64.9		98	No		N/A
P17	ACE inhibitor, beta-blocker	100/70	No		1.6		93	No		Yes

*ACE *angiotensin converting enzyme, *ARB* angiotensin II receptor blocker, *BP*  blood pressure, *CCB* Calcium channel blocker, *GFR* glomerular filtration rate, *N/A *data not available, *U-albcre* urine albumin to creatinine ratio. Blood pressure level and urine albumin to creatinine ratio are values determined during the last year at the time of the study.

### Mitochondrial disease manifestations

Eight patients (47%) had a diagnosis of mitochondrial myopathy. Cardiomyopathy was diagnosed in six patients (35%). Four patients (24%) had a diagnosis of epilepsy or had a history of epileptic seizures. Coronary artery disease was diagnosed in three patients (18%); three patients had a history of stroke-like episodes (18%).

### Clinical characteristics by autoantibody status

No clear differences in diabetes treatment, cardiovascular risk factors, or mitochondrial disease manifestations were apparent between the two patients with marginally positive autoantibodies and antibody-negative patients. However, because autoantibody testing was incomplete and only two patients had low-titre positivity, no formal comparison was performed.

### Prevalence and incidence estimates

Based on the number of patients with mitochondrial DM alive and residing in Southwest Finland on the prevalence date, the prevalence of monogenic DM in the region of Southwest Finland in the end of 2022 was 15/100,000 (95% confidence interval 8.4–24) and the prevalence of mitochondrial DM was 2.7/100,000 (95% confidence interval 0.6–7.2). In the years 2000–2022, a total of 17 patients were identified in the cohort; however, one patient had been diagnosed with mitochondrial diabetes before the incidence period and was therefore excluded from the incident case count. Consequently, a total of 16 new cases of mitochondrial DM were diagnosed during 2000–2022, which corresponds to an average of 0.7 new cases per year giving an annual incidence was 0.14/ 100,000 (95% confidence interval 0.03–3.7).

## Discussion

In this retrospective, observational single-centre study on mitochondrial DM in Southwestern Finland, we looked in detail at the clinical features and treatment of this condition, as well as the prevalence and incidence of mitochondrial DM in this region.

The age at diagnosis of DM varied considerably (11–60 years), but on average, DM was usually diagnosed at a relatively young age (35 years) among these patients, which is in line with previous studies [10]. The diagnostic delay from the diagnosis of DM to the identification of its mitochondrial aetiology was on average five years, whereas a longer diagnostic delay of ten years has been reported previously [15]. None of the patients in this study were overweight at the time of DM diagnosis. According to previous studies, a normal or low BMI is indeed typical of patients with mitochondrial DM, while those with regular type 2 DM are often overweight [14]. Normal BMI does not distinguish mitochondrial diabetes from type 1 diabetes, but the combination of low/normal BMI, negative or low-titre autoantibodies, preserved C-peptide, maternal inheritance, and sensorineural hearing loss should raise suspicion of mitochondrial diabetes.

In the present study, T1D -associated autoantibodies were not systematically tested, as the initial diagnosis was typically T2D. Of the 11 patients tested for GAD antibodies, nine were negative, while two had marginally elevated levels (11 and 52 IU/mL). These low-titre results may represent nonspecific or borderline positivity, coincidental autoimmunity, or rarely overlapping autoimmune diabetes. Seronegative type 1 diabetes remains a differential diagnosis in lean young patients, but in the present cohort the presence of pathogenic mtDNA variants and extra-pancreatic mitochondrial manifestations supported mitochondrial diabetes. Previous studies have reported GAD antibodies to be rare among patients with the m.3243A>G variant [16–18].

A large proportion of the patients in our study (82%) were diagnosed with hearing loss, which is consistent with previous literature reporting sensorineural hearing loss being common manifestation of the same underlying mitochondrial disorder and is therefore an important diagnostic clue in patients with suspected mitochondrial diabetes [7, 10, 18]. As the onset of hearing loss in insidious, the precise time of onset is not always easy to determine. Previous studies suggest that in mitochondrial disease related to the m.3243A>G variant, both sensorineural hearing loss and diabetes have their onset in adult age. The mean age of onset of diabetes has been reported to be 38 years in two studies on patients with m.3243A>G and the age of onset of hearing loss to be 41 years [19]. The average age at which patients started using a hearing aid (38 years, range 11–57 years) is also in line with previous studies [20].

Renal failure is often associated with mitochondrial DM [4]; this was also the case in 35% of patients in this data set. Renal involvement in mitochondrial disease may also occur independently of diabetes. Thus, albuminuria or reduced eGFR in patients with mitochondrial diabetes may reflect mitochondrial nephropathy, diabetic kidney disease, hypertension-related kidney disease, or overlapping mechanisms. This distinction is clinically meaningful but is challenging to establish retrospectively.

Treatment of mitochondrial DM should be individualized according to glycaemic severity, residual beta-cell function, renal function, comorbidities, and the overall mitochondrial disease phenotype. Insulin is often the primary treatment if there is insulin deficiency [21]. Lifestyle modifications in addition to oral medications may be sufficient in some cases [22]. Metformin is commonly suggested best avoided because it inhibits mitochondrial respiratory chain complex I and may increase the risk of lactic acidosis, particularly in patients with renal impairment or severe mitochondrial myopathy. However, anecdotal evidence suggests it may be useful and well tolerated in particularly overweight patients [23]. Moreover, a recent expert consensus agreed that metformin could be safely used in the management of primary mitochondrial diseases [24]. Overall, we suggest that the potential use of metformin should be based on individualized risk-benefit assessment. SGLT2 inhibitors, GLP-1 receptor agonists, and DPP-4 inhibitors may offer therapeutic benefits in mitochondrial DM, but data supporting their use remain limited. In our data, four patients were using a gliptin, and three patients were using a SGLT2 inhibitor (dapagliflozin or empagliflozin) in addition to insulin, and one was currently using metformin. Treatment decisions were based on individualized assessment by experienced diabetes physicians and no causal conclusions about treatment efficacy can be drawn from this retrospective cohort. To date, there are little published data on SGLT2 inhibitors in the treatment of mitochondrial DM. A case series of three patients treated with empagliflozin with no reported adverse effects has been published [25]. However, no safety concerns or adverse effects related to these medications were noted in patient records. In our data, seven patients (41%) had used metformin at some point, typically before they were diagnosed with a mitochondrial disorder. Disease-specific treatment guidelines for mitochondrial diabetes have been lacking [26]. However, publications provide overviews on the treatment of mitochondrial diabetes including metabolic control and more novel diabetes medications. In addition, a treatment algorithm has recently been proposed [25, 27].

In mitochondrial DM, the treatment goals and diagnostic cutoffs for glucose levels are the same as in other forms of DM [25]. In at least one study, patients with mitochondrial DM reached or approached the treatment goal (HbA1c value below 7.0% o 53 mmol/mol) with oral antidiabetic drugs alone, excluding metformin [25]. However, in this study, only six (35%) patients had HbA1c below 7.0% (53 mol/mol).

Most patients (82%) were using insulin, and the average time from diagnosis to starting insulin was 3.5 years. In a previous study, insulin treatment was started on average two years after diagnosis [4]. In addition to metformin, statins have been advised to be avoided in MIDD patients because of muscle related side effects [14]. There are few publications on statin treatment in patients with mitochondrial disease. Despite concerns of heightened risk of statin induced myopathy [28], there are also reports of safe treatment with statins in this patient group [29]. In our data, seven out of nine statin users experienced some form of statin-associated muscle symptoms. With the current available evidence, the benefits and risks of using statins must be weighed individually.

Available C-peptide values suggested residual endogenous insulin secretion in several patients, supporting the concept of progressive beta-cell dysfunction rather than absolute insulin deficiency at diagnosis. However, C-peptide measurements were not available for all patients and were not standardized; therefore, no firm conclusions regarding treatment choice or metabolic control can be drawn.

Recently, the minimum prevalence of adult mtDNA related mitochondrial disease in Southwestern Finland was estimated at 9.2/100 000, whilst the prevalence of m.3243A>G, the most common pathogenic mtDNA variant underlying mitochondrial DM, was estimated at 4.2/100 000 [12]. The estimated prevalence for mitochondrial DM reached here (2.7/100 000) shows that DM is a common clinical feature of mitochondrial disease. In this study, only one of the patients had another pathogenic mtDNA variant, the m.3260A>G. This variant is in the same mitochondrial tRNA gene, *MT-TL1*, as the more common m.3243A>G variant, and has been associated with multiple mitochondrial disease phenotypes including myopathy, cardiomyopathy, and the MELAS syndrome [22, 23].

Potential remaining undiagnosed cases are always a possibility. However, our recent prevalence estimates of diagnosed mitochondrial disease are well in line with figures published by internationally recognized expert centres suggesting that considerable underestimation of mitochondrial DM is unlikely [12]. All patients with a diagnosis of mitochondrial DM received genetic counselling that includes pedigree analysis. Several patients included in this study had a positive family history of mitochondrial disease (Table 1). However, as this work describes clinical diagnostics, we were not able to assess the prevalence of DM in family members that were not patients at TUH. Studies suggest that the m.3243A>G variant arises not infrequently *de novo* [30, 31], so it is likely that the seemingly unrelated cases really are such.

As to the weaknesses and strengths of the study, relatively small number of patients possibly limits the generalizability of the results. As it is fairly typical for retrospective studies, some data were missing for the subjects. Weight and C-peptide values at the time of diagnosis were not available for all patients. For the majority of patients, data on possible diabetic neuropathy was not available.

Kidney involvement was obtained from routine medical records rather than assessed according to a predefined study protocol. The timing of microvascular complications and pre-diabetes proteinuria screening could not be reliably assessed from the available retrospective records. Also, the differentiation of retinopathy and neuropathy from mitochondrial disease-related manifestations was not always possible retrospectively. However, because the study was conducted in a single centre and comprehensive electronic patient data for a long period were available, we could collect and analyse detailed data for the patients. Moreover, we were able to give reliable estimates on the prevalence and incidence of mitochondrial DM for the area of Southwestern Finland. Despite the distinct genetic features of the Finnish population in comparison with other Europeans [24, 25], previous studies suggest that the prevalence of mitochondrial disease in Southwest Finland is similar to that observed in the UK [12, 26].

There is typically several years’ delay in identifying DM as mitochondrial in aetiology. The likely reasons for this are the rarity of mitochondrial disease and the somewhat diverse clinical presentations. A long diagnostic delay is recognised as a common feature in rare diseases [32]. Important clinical clues that may indicate the possibility of mitochondrial DM are non-autoimmune DM with onset in early adulthood, low or normal BMI, and sensorineural hearing loss that usually develops at a young age, commonly leading to the use of a hearing aid. Some patients with mitochondrial DM may also have a broader, syndromic manifestation that may include e.g. myopathy, epilepsy, ataxia or stroke-like episodes; these are particularly associated with the m.3243A>G variant [5]. Although the treatment of mitochondrial diseases is currently symptomatic, the correct diagnosis is important not only because of the specific features of genetic counselling and mitochondrial DM treatment, but also because of the severe and even life-threatening complications that may be associated with the disease. These include stroke-like episodes [33], acute paralytic ileus [34] and sudden cardiac death [35].

In conclusion, non-autoimmune DM with onset in young adulthood, especially if accompanied by sensorineural hearing loss, merits investigations to detect possible mitochondrial DM. This should be considered already at the level of primary diabetes care. The correct recognition of the mitochondrial origin of diabetes has important therapeutic implications. Many patients required insulin therapy during follow-up, which is consistent with progressive beta-cell dysfunction described in mitochondrial diabetes. Early recognition of mitochondrial diabetes is important for individualized treatment, avoidance of potentially harmful therapies, genetic counselling, and surveillance for renal, auditory, cardiac, neurological, and ophthalmological manifestations.

## Data Availability

Individual patient data is not available. Anonymised aggregate data is available from the corresponding author upon reasonable request.

## References

[1] Ng YS, Bindoff LA, Gorman GS, Klopstock T, Kornblum C, Mancuso M, et al. Mitochondrial disease in adults: recent advances and future promise. Lancet Neurol. 2021;20:573–84. 10.1016/S1474-4422(21)00098-3. 34146515 10.1016/S1474-4422(21)00098-3

[2] Gorman GS, Chinnery PF, DiMauro S, Hirano M, Koga Y, McFarland R, et al. Mitochondrial diseases. Nat Rev Dis Primers. 2016;2:16080. 10.1038/nrdp.2016.80. 27775730 10.1038/nrdp.2016.80

[3] Goto Y, Nonaka I, Horai S. A mutation in the tRNALeu(UUR) gene associated with the MELAS subgroup of mitochondrial encephalomyopathies. Nature. 1990;348:651–3. 10.1038/348651a0. 2102678 10.1038/348651a0

[4] Murphy R, Turnbull DM, Walker M, Hattersley AT. Clinical features, diagnosis and management of maternally inherited diabetes and deafness (MIDD) associated with the 3243A>G mitochondrial point mutation. Diabet Med. 2008;25:383–99. 10.1111/j.1464-5491.2008.02359.x. 18294221 10.1111/j.1464-5491.2008.02359.x

[5] Nesbitt V, Pitceathly RDS, Turnbull DM, Taylor RW, Sweeney MG, Mudanohwo EE, et al. The UK MRC mitochondrial disease patient cohort study: Clinical phenotypes associated with the m.3243A>G mutation–implications for diagnosis and management. J Neurol Neurosurg Psychiatry. 2013;84:936–8. 10.1136/jnnp-2012-303528. 23355809 10.1136/jnnp-2012-303528

[6] Pavlakis SG, Phillips PC, DiMauro S, De Vivo DC, Rowland LP. Mitochondrial myopathy, encephalopathy, lactic acidosis, and strokelike episodes: A distinctive clinical syndrome. Ann Neurol. 1984;16:481–8. 10.1002/ana.410160409. 6093682 10.1002/ana.410160409

[7] van den Ouweland JMW, Lemkes HHPJ, Trembath RC, Ross R, Velho G, Cohen D, et al. Maternally inherited diabetes and deafness is a distinct subtype of diabetes and associates with a single point mutation in the mitochondrial tRNA Leu(UUR) gene. Diabetes. 1994;43:746–51. 10.2337/diab.43.6.746. 7910800 10.2337/diab.43.6.746

[8] van den Ouweland JMW, Lemkes HHPJ, Ruitenbeek W, Sandkuijl LA, de Vijlder MF, Struyvenberg PAA, et al. Mutation in mitochondrial tRNALeu(UUR) gene in a large pedigree with maternally transmitted type II diabetes mellitus and deafness. Nat Genet. 1992;1:368–71. 10.1038/ng0892-368. 1284550 10.1038/ng0892-368

[9] Lindroos MM, Majamaa K, Tura A, Mari A, Kalliokoski KK, Taittonen MT, et al. m.3243A>G Mutation in mitochondrial DNA leads to decreased insulin sensitivity in skeletal muscle and to progressive β-cell dysfunction. Diabetes 2009;58:543–9. 10.2337/db08-0981 10.2337/db08-0981 PMC2646052 19073775

[10] Guillausseau P-J, Massin P, Dubois-LaForgue D, Timsit J, Virally M, Gin H, et al. Maternally inherited diabetes and deafness: A multicenter study. Ann Intern Med. 2001;134:721–8. 10.7326/0003-4819-134-9_Part_1-200105010-00008. 11329229 10.7326/0003-4819-134-9_part_1-200105010-00008

[11] Martikainen MH, Rönnemaa T, Majamaa K. Prevalence of mitochondrial diabetes in southwestern Finland: a molecular epidemiological study. Acta Diabetol. 2013;50:737–41. 10.1007/s00592-012-0393-2. 22492248 10.1007/s00592-012-0393-2

[12] Martikainen MH, Majamaa K. Incidence and prevalence of mtDNA-related adult mitochondrial disease in Southwest Finland, 2009–2022: an observational, population-based study. BMJ Neurol Open. 2024;6:e000546. 10.1136/bmjno-2023-000546. 38361968 10.1136/bmjno-2023-000546 PMC10868302

[13] Massin P, Dubois-Laforgue D, Meas T, Laloi-Michelin M, Gin H, Bauduceau B, et al. Retinal and renal complications in patients with a mutation of mitochondrial DNA at position 3,243 (maternally inherited diabetes and deafness). A case–control study. Diabetologia. 2008;51:1664–70. 10.1007/s00125-008-1073-1. 18581092 10.1007/s00125-008-1073-1

[14] Naing A, Kenchaiah M, Krishnan B, Mir F, Charnley A, Egan C, et al. Maternally inherited diabetes and deafness (MIDD): Diagnosis and management. J Diabetes Complications. 2014;28:542–6. 10.1016/j.jdiacomp.2014.03.006. 24746802 10.1016/j.jdiacomp.2014.03.006

[15] Zheng S, Wang J, Sun M, Wang P, Shi W, Zhang Z, et al. The clinical and genetic characteristics of maternally inherited diabetes and deafness (MIDD) with mitochondrial m.3243A > G mutation: A 10-year follow‐up observation study and literature review. Clin Case Rep. 2024;12. 10.1002/ccr3.8458. 10.1002/ccr3.8458 PMC10834381 38314188

[16] Taniyama M, Kasuga A, Suzuki Y, Ozawa Y, Handa M, Kobayashi A, et al. Absence of antibodies to ICA512/IA-2in NIDDM patients with the mitochondrial DNA bp 3243 mutation. Diabetes Care. 1997;20:905–6. 10.2337/diacare.20.5.905. 9135964 10.2337/diacare.20.5.905

[17] Kobayashi T, Oka Y, Katagiri H, Falorni A, Kasuga A, Takei I, et al. Association between HLA and islet cell antibodies in diabetic patients with a mitochondrial DNA mutation at base pair 3243. Diabetologia. 1996;39:1196–200. 10.1007/BF02658506. 8897007 10.1007/BF02658506

[18] Yang M, Xu L, Xu C, Cui Y, Jiang S, Dong J, et al. The mutations and clinical variability in maternally inherited diabetes and deafness: An analysis of 161 patients. Front Endocrinol (Lausanne). 2021;12. 10.3389/fendo.2021.728043. 10.3389/fendo.2021.728043 PMC8654930 34899594

[19] Majamaa K, Kärppä M, Moilanen JS. Neurological manifestations in adult patients with the m.3243A>G variant in mitochondrial DNA. BMJ Neurol Open. 2024;6:e000825. 10.1136/bmjno-2024-000825. 39324021 10.1136/bmjno-2024-000825 PMC11423728

[20] Uimonen S, Moilanen JS, Sorri M, Hassinen IE, Majamaa K. Hearing impairment in patients with 3243A→G mtDNA mutation: Phenotype and rate of progression. Hum Genet. 2001;108:284–9. 10.1007/s004390100475. 11379873 10.1007/s004390100475

[21] Ng YS, Lim AZ, Panagiotou G, Turnbull DM, Walker M. Endocrine manifestations and new developments in mitochondrial disease. Endocr Rev. 2022;43:583–609. 10.1210/endrev/bnab036. 35552684 10.1210/endrev/bnab036 PMC9113134

[22] Yeung RO, Hannah-Shmouni F, Niederhoffer K, Walker MA. Not quite type 1 or type 2, what now? Review of monogenic, mitochondrial, and syndromic diabetes. Rev Endocr Metab Disord. 2018;19:35–52. 10.1007/s11154-018-9446-3. 29777474 10.1007/s11154-018-9446-3

[23] Schaefer AM, Walker M, Turnbull DM, Taylor RW. Endocrine disorders in mitochondrial disease. Mol Cell Endocrinol. 2013;379:2–11. 10.1016/j.mce.2013.06.004. 23769710 10.1016/j.mce.2013.06.004 PMC3820028

[24] De Vries MC, Brown DA, Allen ME, Bindoff L, Gorman GS, Karaa A, et al. Safety of drug use in patients with a primary mitochondrial disease: An international Delphi-based consensus. J Inherit Metab Dis. 2020;43:800–18. 10.1002/jimd.12196. 32030781 10.1002/jimd.12196 PMC7383489

[25] Yeung RO, Al Jundi M, Gubbi S, Bompu ME, Sirrs S, Tarnopolsky M, et al. Management of mitochondrial diabetes in the era of novel therapies. J Diabetes Complications. 2021;35:107584. 10.1016/j.jdiacomp.2020.107584. 32331977 10.1016/j.jdiacomp.2020.107584 PMC7554068

[26] Chanoine J-P, Thompson DM, Lehman A. Diabetes associated with maternally inherited diabetes and deafness (MIDD): From pathogenic variant to phenotype. Diabetes. 2025;74:153–63. 10.2337/db24-0515. 39556456 10.2337/db24-0515 PMC11755681

[27] Chaudhry A, Thompson DM, Chanoine J. Diabetes management in maternally inherited diabetes and deafness (< scp>MIDD): A review and a proposed treatment algorithm. Diabetes Obes Metab. 2026;28:826–39. 10.1111/dom.70240. 41145374 10.1111/dom.70240 PMC12803549

[28] Mollazadeh H, Tavana E, Fanni G, Bo S, Banach M, Pirro M, et al. Effects of statins on mitochondrial pathways. J Cachexia Sarcopenia Muscle. 2021;12:237–51. 10.1002/jcsm.12654. 33511728 10.1002/jcsm.12654 PMC8061391

[29] Hannah-Shmouni F, Al-Sarraf A, Frohlich J, Mezei MM, Sirrs S, Mattman A. Safety of statin therapy in patients with mitochondrial diseases. J Clin Lipidol. 2013;7:182. 10.1016/j.jacl.2012.08.003. 23415441 10.1016/j.jacl.2012.08.003

[30] Pierron D, Rocher C, Amati-Bonneau P, Reynier P, Martin-Négrier M-L, Allouche S, et al. New evidence of a mitochondrial genetic background paradox: Impact of the J haplogroup on the A3243G mutation. BMC Med Genet. 2008;9:41. 10.1186/1471-2350-9-41. 18462486 10.1186/1471-2350-9-41 PMC2409300

[31] de Laat P, Janssen MCH, Alston CL, Taylor RW, Rodenburg RJT, Smeitink JAM. Three families with ‘de novo’ m.3243A>G mutation. BBA Clin. 2016;6:19–24. 10.1016/j.bbacli.2016.04.007. 27331024 10.1016/j.bbacli.2016.04.007 PMC4900294

[32] Faye F, Crocione C, Anido de Peña R, Bellagambi S, Escati Peñaloza L, Hunter A, et al. Time to diagnosis and determinants of diagnostic delays of people living with a rare disease: results of a Rare Barometer retrospective patient survey. Eur J Hum Genet. 2024;32:1116–26. 10.1038/s41431-024-01604-z. 38755315 10.1038/s41431-024-01604-z PMC11369105

[33] Mickelsson N, Hirvonen J, Martikainen MH. Clinical features and treatment of stroke-like episodes in mitochondrial disease: a cohort-based study. J Neurol. 2025;272:47. 10.1007/s00415-024-12745-y. 10.1007/s00415-024-12745-y PMC11638336 39666093

[34] Ng YS, Feeney C, Schaefer AM, Holmes CE, Hynd P, Alston CL, et al. Pseudo-obstruction, stroke, and mitochondrial dysfunction: A lethal combination. Ann Neurol. 2016;80:686–92. 10.1002/ana.24736. 27453452 10.1002/ana.24736 PMC5215534

[35] Ng YS, Grady JP, Lax NZ, Bourke JP, Alston CL, Hardy SA, et al. Sudden adult death syndrome in m.3243A>G-related mitochondrial disease: an unrecognized clinical entity in young, asymptomatic adults. Eur Heart J 2016;37:2552–9. 10.1093/eurheartj/ehv306. 10.1093/eurheartj/ehv306 PMC5008417 26188002

